# Medication use for the treatment of diabetes in obese individuals

**DOI:** 10.1007/s00125-017-4288-1

**Published:** 2017-05-14

**Authors:** John P. H. Wilding

**Affiliations:** Obesity and Endocrinology Research, Institute of Ageing and Chronic Disease, Clinical Sciences Centre, University of Liverpool, Longmoor Lane, Liverpool, L9 7AL UK

**Keywords:** Diabetes, GLP-1 agonists, Lorcaserin, Naltrexone/bupropion, Obesity, Orlistat, Phentermine/topiramate, Review, SGLT2

## Abstract

Obesity is a major cause of type 2 diabetes and may complicate type 1 diabetes. Weight loss for obese individuals with diabetes has many health benefits, often leads to improvement in glucose control and sometimes, in type 2 diabetes, near normalisation of abnormal glucose metabolism. Weight loss is difficult to maintain and attempts to lose weight may be undermined by some diabetes treatments such as sulfonylureas, thiazolidinediones and insulin. Whilst lifestyle support should be the primary approach to aid individuals who wish to lose weight, pharmacological approaches can also be considered. These include choosing glucose-lowering drugs or drug combinations that are weight neutral or result in weight loss or prescribing drugs that are specifically approved as anti-obesity medication. Given that some of the newer glucose-lowering medications that cause weight loss, such as glucagon-like peptide-1 receptor agonists (GLP-1 RAs) and sodium–glucose cotransporter 2 inhibitors (SGLT2i), are also being used or considered for use as anti-obesity drugs, it seems that the distinction between glucose-lowering medication and weight loss medication is becoming blurred. This review discusses the main pharmacological approaches that can be used to support weight loss in individuals with diabetes.

## Introduction

Type 2 diabetes is an obesity-related disease. The recent increase in the prevalence of type 2 diabetes worldwide has largely mirrored the rise in obesity and, although the precise pathways leading to diabetes are yet to be fully elucidated, the current understanding of its pathophysiology recognises the role of increased adipose tissue and ectopic fat deposition in causing both insulin resistance and progressive beta cell failure [1]. In people with established diabetes, the severity of obesity may contribute to the development of complications [2], although some studies suggest protection from cardiovascular disease in those who are overweight [3]. Short-term studies of energy restriction [4, 5] and longer term data from individuals who have lost weight after bariatric surgery have shown that weight loss can lead to improvement of both insulin resistance and impaired beta cell function. As a result, individuals may experience diabetes remission, which can be long lasting if weight loss is maintained [6]. The amount of weight loss needed to provide benefit to people with type 2 diabetes is about 3% but additional benefit is seen up to at least 15%, possibly more [7]. Despite this evidence, most individuals with type 2 diabetes receive little support to help them manage their weight and the use of some glucose-lowering medications may result in further weight gain. It should also be remembered that a significant proportion of individuals with type 1 diabetes are also obese and that weight gain is a frequent consequence of intensive insulin treatment [8]. The question, therefore, arises as to how best to support individuals with diabetes to enable the management of their weight.

Lifestyle intervention, with its three core components of diet, physical activity and behaviour change [9], remains the essential first step in achieving weight loss. However, the reality is that, even when offered in the context of a clinical trial, such as the Action for Health in Diabetes (Look AHEAD) study [10], intensive lifestyle intervention only results in modest sustained weight loss (∼4 kg difference from control participants) and weight regain is common. It is, however, important to note that such interventions do improve vascular risk factors such as lipids, blood pressure and microalbuminuria, at least in the short term, and also improve quality of life [11]. The primary analysis of the Look AHEAD study did not show any vascular benefit of lifestyle intervention, although vascular risk factors were improved, mostly in those participants with greater weight loss [12]. Bariatric surgery is effective for weight loss, but currently only a minority of individuals are able to access this treatment option [6]. This review will focus on the use of medication for weight management, and will consider the appropriate use of glucose-lowering drugs that result in weight loss, or of treatments specifically indicated to aid weight loss and weight maintenance, to support individuals with diabetes in their efforts to lose weight. The focus will largely be placed on type 2 diabetes but consideration will also be given to type 1 diabetes where appropriate evidence exists.

## Glucose-lowering diabetes medication and weight change

Current ADA/EASD guidelines for the treatment of type 2 diabetes [13] emphasise the importance of lifestyle-promoted weight management for obese and overweight individuals, and support an individualised choice of medication to favour medicines that are ‘weight neutral’ or result in weight loss for individuals in whom weight loss is considered a priority. In general, sodium–glucose cotransporter 2 inhibitors (SGLT2i) and glucagon-like peptide (GLP)-1 receptor agonists (GLP-1 RAs) result in an average weight loss of about 2–3 kg when used at the approved doses, whilst metformin, alpha-glucosidase inhibitors and dipeptidyl peptidase-4 inhibitors (DPP-IVi) are weight neutral (although some trials report a small mean weight loss of 0.5–1 kg) and sulfonylureas, glinides, thiazolidinediones and insulin result in weight gain [14], although individual responses can vary considerably.

### Mechanisms underlying the effect of glucose-lowering medication on weight in type 2 diabetes

The mechanisms leading to weight gain with use of insulin (and probably also insulin secretagogues) in individuals with high blood glucose include a reduction in energy loss via glycosuria, the anabolic effects of insulin and an associated increase in food intake [15]; whilst the anabolic effects of insulin are beneficial in individuals who are relatively insulin deficient, in whom catabolic processes are highly active, for obese individuals this may be detrimental, contributing to a cycle of weight gain and worsening insulin resistance [16]. Thiazolidinedione-associated weight gain appears to be related to an increase in adipose tissue deposition in subcutaneous depots [17], although this class of drugs may also decrease visceral fat deposition [18]. Thus, it is possible that the weight gain associated with thiazolidinedione use may be less harmful than that with other drug classes.

The glucose-lowering drugs that result in weight loss do so by contributing to a negative energy balance. For example, the SGLT2i, which inhibit renal glucose transport, induce loss of about 75 g (approximately 1200 kJ [300 kcal]) of glucose in the urine. However, the weight loss resulting from use of SGLT2i is less than expected, possibly because of a compensatory increase in food intake [19]. As an example, the GLP-1 RAs reduce appetite and, thus, food intake when administered to humans, and although nausea and delayed gastric emptying are adverse effects of these drugs, the reduction in appetite seems to occur independently of these side effects. Instead, it is likely to result from a central mechanism since GLP-1 receptors are present in the brainstem, hypothalamus and other brain regions involved in the regulation of energy balance and administration of GLP-1 or its analogues into the relevant brain areas reduces food intake in rodents [20, 21].

Intra-class difference may also exist within groups of drugs in relation to their effects on weight loss. Important examples here are the GLP-1 RAs: head-to-head studies suggest that liraglutide is most effective for weight loss, whereas weight loss is somewhat less with albiglutide, perhaps because the albumin component of this drug limits its ability to reach the central nervous system [22]. There are also subtle differences between different basal insulins, with less weight gain reported for insulin detemir compared with NPH insulin, but the effect size is small and of doubtful clinical significance [23].

### Combination therapy for weight management in type 2 diabetes

Most individuals with diabetes require combination therapy as the condition progresses. Given that most are initially started on metformin, the most suitable combinations for dual therapy where weight loss is important are metformin + SGLT2i and metformin + GLP-1 RA. If triple therapy is needed, then the combination of metformin + SGLT2i + DPP-IVi would seem suitable [13]. There is limited data on the combination of metformin + SGLT2i + GLP-1 RA but the results of the DURATION 8 study showed that a combination of dapagliflozin (an SGLT2i) once daily and exenatide (a GLP-1 RA) once weekly on a background of metformin treatment resulted in a 2% reduction in HbA_1c_ and a weight loss of 3.4 kg after 28 weeks of administration; importantly, these beneficial changes were greater following combined therapy than when these drugs were used in monotherapy [24].

Weight gain is a significant problem for many who are treated with insulin. Nonetheless, despite the fact that both SGLT2i and GLP-1 RAs have been shown to reduce insulin requirements, improve glycaemic control and mitigate weight gain when added to therapy for insulin-treated individuals, current recommendations support continuation of metformin with insulin use, unless this is contraindicated [13]. From a practical perspective it would also seem logical to continue SGLT2i and GLP-1 RAs in patients who are already taking these medications and are commencing basal insulin therapy, although there are limited trial data to support this approach [25].

SGLT2i and GLP-1 RAs may have other advantages when used in diabetes treatment; there is emerging evidence that they may reduce visceral, particularly hepatic, fat deposition [26]. Both classes of drugs also reduce blood pressure via both weight-dependent and weight-independent mechanisms [27]. Although the Evaluation of Lixisenatide in Acute Coronary Syndrome (ELIXA) trial was neutral for an effect on cardiovascular outcomes, the Empagliflozin, Cardiovascular Outcomes, and Mortality in Type 2 Diabetes (EMPA-REG) OUTCOME trial [28, 29], the Liraglutide Effect and Action in Diabetes: Evaluation of Cardiovascular Outcome (LEADER) trial [30] and The Trial to Evaluate Cardiovascular and Other Long-term Outcomes With Semaglutide in Subjects With Type 2 Diabetes (SUSTAIN 6) [31] showed improvements in cardiovascular and renal outcomes in individuals at high-risk of cardiovascular disease that were treated with these agents.

### The impact of glucose-lowering drugs on weight in type 1 diabetes

Given that the problem of weight gain with intensive insulin treatment is known, the option of adding drugs that might attenuate this to treatment regimens for type 1 diabetes has been investigated in a number of trials. There is some evidence to support metformin use to mitigate weight gain in type 1 diabetes, although the weight change with metformin has been found to be modest [32]. Similarly, in type 1 diabetes cohorts, trials with GLP-1 RAs have been disappointing, resulting in only modest weight loss with a negligible effect on glucose [33]. There has also been considerable interest in the use of SGLT2i in type 1 diabetes but initial enthusiasm has been dampened by the recognition that this class of drugs may be associated with the development of ketoacidosis in susceptible individuals [34]. Hence, the results of several ongoing trials in this area are awaited with interest.

## Currently available drugs for obesity and their use in diabetes

When clinically appropriate, it is important to consider the potential role of medicines that are approved for weight management as additional treatments for people with diabetes who wish to lose weight. The use of drugs for obesity treatment has been a controversial topic and a number of agents have been withdrawn following their approval, including dexfenfluramine (links to cardiac valvular disorders), sibutramine (increased risk of adverse cardiovascular events) and rimonabant (mood disorders including suicidality) [9]. However, orlistat has been available for many years with a reasonable safety record and long-term data for up to 4 years treatment. Several new agents/therapeutic strategies have recently been approved for use in the USA and elsewhere, although not all are available in Europe. These are: (1) the GLP-1 RA liraglutide given at a higher dose of 3 mg (a maximum dose of 1.8 mg is prescribed for diabetes treatment); (2) the 5-hydroxytryptamine_2C_ (5-HT_2C_) serotonin receptor agonist lorcaserin; (3) combination treatment of the centrally acting sympathomimetic phentermine with topiramate; and (4) combined treatment with the μ-opioid antagonist naltrexone plus the noradrenaline (norepinephrine) and dopamine reuptake inhibitor bupropion. Phentermine monotherapy is also approved for short-term use only, as is the use of other related compounds (such as diethylpropion), on the basis of limited data and will not be discussed further. A summary of the mode of action of each drug/drug combination, dosing, the effects in type 2 diabetes on body weight, HbA_1c_, blood pressure, blood lipids, and the most important contraindications and adverse events are provided in Table 1.

**Table 1:** Drugs for obesity and their effects in type 2 diabetes

Drug/ combination	Mechanism of action	Route of administration/dosing	Weight change in placebo arm of T2DM trials at 1 year: absolute (kg)/% with 5% weight loss	Weight change in T2DM at 1 year: absolute (kg)/% with 5% weight loss	Effects on HbA_1c_ (%/mmol/mol)	Effects on other health risk factors	Limitations/main adverse effects
Orlistat [35]	Intestinal lipase inhibitor	p.o. 120 mg t.d.s. (60 mg t.d.s. OTC)	−1.4 kg/NR	−3.8 kg/23%	−0.69% 8 mmol/mol	Systolic BP: −1.8 mmHg LDL-cholesterol: −1.8%	Oily/fatty stools Anal leakage Reductions in fat-soluble vitamins
Liraglutide [36]	GLP-1 RA	s.c. titrate in 0.6 mg weekly increments to 3 mg o.d.	−2.2 kg/21.4%	−6.0 kg/54.3%	−1.3% 14 mmol/mol	Systolic BP: −1 mmHg LDL-cholesterol: −3.1%	Nausea/vomiting Gallstones Pancreatitis
Lorcaserin [37]	5-HT_2C_ receptor agonist	p.o. 10 mg b.d.	−1.6 kg/16.1%	−5.0 kg/37.5%	−0.9% 10 mmol/mol	Systolic BP: −0.8 mmHg (NS) LDL-cholesterol: +4.2 (NS)	Headache Back pain Nasopharyngitis Possible cardiac valvulopathy
Naltrexone/ bupropion [38]	μ-opioid antagonist/dopamine and noradrenaline reuptake inhibitor	8 mg/90 mg o.d., increasing over 2 weeks to 16 mg/180 mg b.d.	−1.8 kg/18.9%	−5.0 kg/44.5%	−0.6% 7 mmol/mol	Systolic BP: +1 mmHg LDL-cholesterol: no change	Nausea Constipation Vomiting Dizziness Dry mouth Increased BP and heart rate
Phentermine/ topiramate [39]	Centrally acting sympathomimetic/anticonvulsant	3.75 mg/23 mg o.d. for 14 days then increase to 7.5 mg/46 mg; dose can be increased to 15 mg/92 mg o.d. if 3% weight loss not achieved after 12 weeks	−2.6 kg/24%	−9.8 kg/70%	−0.4% 4 mmol/mol	Systolic BP: −3.2 mmHg LDL-cholesterol: −2.8%	Increased heart rate Teratogenic Nausea Dry mouth Constipation Paraesthesia

These data are from separately published studies and, therefore, are not intended to indicate comparative efficacy

Orlistat, liraglutide and naltrexone/bupropion are approved in the USA and European Union; lorcaserin and phentermine/topiramate are only approved in the USA

^a^Changes in HbA_1c_ in diabetes depend on many factors, including baseline HbA_1c_ levels, duration of disease and intensity of lifestyle intervention

b.d., twice daily; NR, not reported; NS, not significant; o.d., once daily; OTC, over the counter; p.o., oral; t.d.s., three times daily

### Important considerations when prescribing drugs for obesity in type 2 diabetes

Drugs for the treatment of obesity should only be offered as an adjunct to a multicomponent lifestyle programme. The precise indications differ slightly between weight-management drugs, but in general they can be considered for individuals with obesity (BMI >30 kg/m^2^) or in those with a BMI >27 kg/m^2^ with significant comorbidity, such as type 2 diabetes. Their effects should be closely monitored and all of the currently approved agents are subject to ‘stopping rules’, which advise that drug treatment should be stopped if a weight loss of 5% or greater is not achieved after 3 months at the maximum tolerated dose or if significant weight regain occurs whilst on treatment. With the exception of orlistat, there are limited published data beyond 2 years of treatment with the aforementioned available weight-management drugs, and somewhat less data for individuals with established type 2 diabetes. Therefore, further research is required in this area to elucidate the optimal therapeutic strategy for weight management in type 2 diabetes.

### Currently available drugs for obesity in individuals with type 1 diabetes

There are no high-quality trials of obesity drugs in individuals with type 1 diabetes. Therefore, although the use of these drugs is not contraindicated in type 1 diabetes, prescription in individuals with this condition should be based on a careful evaluation and discussion of the potential risks and benefits. Investigations of the effects of combined therapy with weight-management agents and glucose-lowering drugs that also cause weight loss is of potential interest, but at present only limited data is available.

## Experimental medicines for obesity/diabetes

There is substantial interest in the development of new treatments to support weight management, particularly in type 2 diabetes, and emerging data from some experimental treatments and novel combinations of existing treatment shows significant recent progress. Therapeutic approaches to support weight loss include the use of drugs that cause energy wastage, for example fat malabsorption with orlistat and urine glucose excretion with SGLT2i. However, the benefits associated with these therapies appear to be limited by a compensatory rise in food intake and, so, combinations of these drugs with anorectic agents are being explored. In general, drugs that increase energy expenditure have proven unsuccessful for inducing weight loss or have unacceptable adverse effects (such historical drugs include the β_3_-adrenoceptor agonists, thyroid hormones and mitochondrial uncouplers, such as dinitrophenol). Thus, most novel approaches currently under investigation include drugs that work by reducing food intake, rather than increasing energy expenditure (as outlined in the text box). However, it must be noted that none of these drugs or drug combinations are currently approved for use and there is limited data to support their use specifically in diabetes.
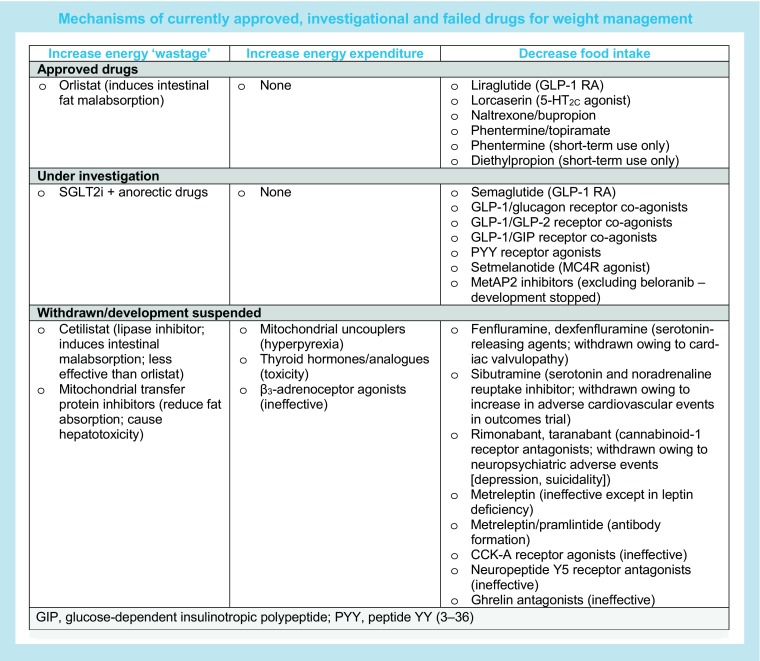


### Phentermine/canagliflozin

As discussed previously, although SGLT2i reduce body weight when used for the treatment of type 2 diabetes, weight loss is less than expected, largely because of a compensatory increase in food intake. It therefore seems logical to combine SGLT2i with anorexigenic drugs. As aforementioned, when used on a metformin background, the combination of dapagliflozin with modified-release exenatide resulted in greater weight loss than either agent alone [24]. Results of a Phase II trial of the combination of phentermine 15 mg with canagliflozin 300 mg in individuals without diabetes were recently reported; the results showed greater weight loss with combination therapy than with use of either agent alone [40]. It will be interesting to see whether this combined therapeutic approach is also effective in individuals with diabetes.

### Melanocortin 4 receptor agonists

The hypothalamic melanocortin-4 receptor (MC4R) plays an important role in the regulation of food intake, as demonstrated by the severe early-onset obesity seen in people with inherited loss-of-function mutations in this receptor. Specific MC4R agonists have been developed and are effective in individuals with MC4R defects [41]; it remains to be seen whether these agents will also be effective for other forms of obesity.

### Methionyl aminopeptidase 2 inhibitors

Methionyl aminopeptidase 2 (MetAP2) is an enzyme that is involved in the removal of *N*-methionine residues from newly synthesised proteins. Irreversible inhibitors of MetAP2, such as beloranib, were originally developed as potential antineoplastic drugs but were subsequently found to induce significant weight loss in clinical trials [42]. However, the development of beloranib was stopped because of an increase in venous thromboembolism in trials of individuals with Prader–Willi syndrome. Thus, whilst this still seems a promising pathway, it remains to be seen whether a drug with an acceptable safety and tolerability profile can be developed for the management of obesity.

### Gut peptides

The satiety cascade begins in the gastrointestinal tract and signals from the gut to the brain that control food intake include nutrients, neural signals and hormones. The hormone GLP-1(7–36)amide, which is already being exploited for treatment of diabetes and obesity, is just one product of the preproglucagon gene; others, including oxyntomodulin and glucagon, also have anorectic effects. Other gut hormones shown to reduce food intake in humans include cholecystokinin (CCK), peptide YY(3–36) (PYY) and pancreatic polypeptide. The stomach also produces the orexigenic peptide ghrelin. The development of agonists (or antagonists in the case of ghrelin) of these peptides has been the focus of much interest [43]. Given the relatively modest effects of individual gut peptides, their redundancy in the system and the fact that native peptides have a short half-life, there has been increasing interest in the development of long-acting analogues, combination treatments and hybrid molecules with effects on more than one receptor to maximise beneficial outcomes [44]. Examples of hybrid drugs include single molecules that act on both GLP-1 and glucose-dependent insulinotropic polypeptide (GIP) receptors, GLP-1 and GLP-2 receptors, or on GLP-1 and glucagon receptors; triple agonists have also been developed [45]. At present little data is available regarding these hybrid therapies but this seems a promising area for future research.

## Summary and conclusion

Given that most type 2 diabetes is obesity-related, it makes sense to favour treatment strategies that promote weight loss. It is also important to consider the use of specific ‘anti-obesity’ treatments to support an individual’s efforts at lifestyle change. Combinations of weight-loss drugs and glucose-lowering agents for obesity/diabetes management, and the use of some drugs in either of these categories for both indications blurs the distinction between obesity and diabetes treatments. For example, SGLT2i and GLP-1 RAs are already available glucose-lowering agents that promote modest reductions in weight and are likely to play a greater role in the management of diabetes in the future, especially given the favourable results of their use in recent cardiovascular outcome trials. On the other hand, novel obesity-specific treatments show promise in diabetes management and, hence, their use in the treatment of diabetes seems likely to increase over time.

## Abbreviations

AHEAD: Action for Health in Diabetes
CCK: Cholecystokinin
DPP-IVi: Dipeptidyl peptidase-4 inhibitors
GLP: Glucagon-like peptide
GLP-1 RA: Glucagon-like peptide-1 receptor agonist
5-HT_2C_: 5-hydroxytryptamine_2C_
MC4R: Melanocortin-4 receptor
MetAP2: Methionyl aminopeptidase 2
PYY: Peptide YY
SGLT2: Sodium–glucose cotransporter 2
SGLT2i: Sodium–glucose cotransporter 2 inhibitors
